# Novel LanT Associated Lantibiotic Clusters Identified by Genome Database Mining

**DOI:** 10.1371/journal.pone.0091352

**Published:** 2014-03-12

**Authors:** Mangal Singh, Dipti Sareen

**Affiliations:** Department of Biochemistry, Panjab University, Chandigarh, India; University of Kansas Medical Center, United States of America

## Abstract

**Background:**

Frequent use of antibiotics has led to the emergence of antibiotic resistance in bacteria. Lantibiotic compounds are ribosomally synthesized antimicrobial peptides against which bacteria are not able to produce resistance, hence making them a good alternative to antibiotics. Nisin is the oldest and the most widely used lantibiotic, in food preservation, without having developed any significant resistance against it. Having their antimicrobial potential and a limited number, there is a need to identify novel lantibiotics.

**Methodology/Findings:**

Identification of novel lantibiotic biosynthetic clusters from an ever increasing database of bacterial genomes, can provide a major lead in this direction. In order to achieve this, a strategy was adopted to identify novel lantibiotic biosynthetic clusters by screening the sequenced genomes for LanT homolog, which is a conserved lantibiotic transporter specific to type IB clusters. This strategy resulted in identification of 54 bacterial strains containing the LanT homologs, which are not the known lantibiotic producers. Of these, 24 strains were subjected to a detailed bioinformatic analysis to identify genes encoding for precursor peptides, modification enzyme, immunity and quorum sensing proteins. Eight clusters having two LanM determinants, similar to haloduracin and lichenicidin were identified, along with 13 clusters having a single LanM determinant as in mersacidin biosynthetic cluster. Besides these, orphan LanT homologs were also identified which might be associated with novel bacteriocins, encoded somewhere else in the genome. Three identified gene clusters had a C39 domain containing LanT transporter, associated with the LanBC proteins and double glycine type precursor peptides, the only known example of such a cluster is that of salivaricin.

**Conclusion:**

This study led to the identification of 8 novel putative two-component lantibiotic clusters along with 13 having a single LanM and 3 with LanBC genes. Putative lantibiotic clusters identified here hold the potential for the discovery of novel lantibiotic(s).

## Introduction

Antibiotic compounds are in use for many decades in treating various bacterial infections and their continuous use has led to the development of drug resistant bacterial strains [1]. MRSA (methicillin resistant *Staphylococcus aureus*), VRE (vancomycin resistant *Enterococci*), MDR-TB (multi drug resistant *Mycobacterium tuberculosis*) and MDR-SP (*Streptococcus pneumoniae*) are some of the antibiotic resistant bacterial strains that are difficult to treat, therefore posing a need for new antimicrobial compounds. Lantibiotics are emerging as more promising drugs against these bacteria. Nisin was the first “lantibiotic” discovered from *Lactococcus lactis* [2] and is being used in food preservation since then due to its broad activity against many food spoiling and pathogenic bacteria such as *Bacillus cereus*, *Listeria monocytogenes*, *Enterococci*, *Staphylococci* and *Streptococci*. More than 40 years of its use in food industry has not led to any significant resistance [3], justifying the need and importance of study on lantibiotics.

Lantibiotics are *Lan*thionine containing antibiotics having a characteristic lanthionine ring responsible for their stability. The lanthionine ring is formed by dehydration and cyclization in the core peptide region of a 50–100 amino acid residues long precursor peptide from which the N-terminal leader peptide is cleaved off. The serine and threonine residues are dehydrated to form didehydroalanine (Dha) and didehydrobutyrine (Dhb) residues, respectively. Following dehydration, cyclization occurs by linking cysteine to Dha or Dhb residues thus forming a thioether linkage called as lanthionine or β-methyllanthionine. Lantibiotics exhibit their antibacterial activity by capturing lipid II precursor of peptidoglycan, thus, inhibiting cell wall formation [4], or by pore formation which leads to leakage and disruption of the membrane potential [5]. Nisin exhibits both of these activities. The leader peptide of lantibiotics helps in the recognition and interaction of lanthipeptide with different enzymes of its biosynthetic machinery [6], [7]. It is the leader peptide which keeps the lantibiotic inactive, when present inside the cell [7], [8] and is also required for its extracellular transport [9], [10]. Lantibiotic biosynthetic machinery is organized into gene clusters, in single or multiple operons encoding the precursor peptide(s), modification enzymes, immunity proteins, a protease and a transporter. Following post-translational modifications, the lanthipeptide is exported outside the cell by an ABC (ATP binding cassette) transporter and the leader peptide is cleaved off by a protease. The cleavage is either concomitant with the export [11] or after the transport, by an extracellular protease that may (e.g. nisin) or may not (e.g. subtilin) be encoded in the cluster [12], [13]. Due to a diversity in the lantibiotic compounds, several classification schemes have been proposed [14]–[17]. The widely used bacteriocin mining tool, BAGEL2 [18] is based upon the classification given by Cotter *et al.*, who have classified the lantibiotics into two major classes. Class I lantibiotics includes lanthionine containing lantibiotics and class II includes small non-lanthionine bacteriocins. Class I lantibiotics are further classified into three subgroups [19]. Type IA lantibiotics like nisin [20] and microbisporicin [21] are modified by two separate enzymes LanB and LanC. LanB is responsible for the dehydration of selective serine and threonine residues in the core peptide region and LanC for cyclization leading to lanthionine formation. Type IA precursor peptides have a highly conserved proline at -2 position, which is a characteristic of LanBC modified precursor peptides. In Type IB clusters, lanthionine formation is carried out by a single LanM protein, which carries out both the dehydration and cyclization. Type IC lantibiotics are also modified by a single protein, LanL [22]. In most of the class I clusters, besides the precursor peptide and lanthionine synthetases, a two-component response regulator system (LanRK) is present, same as in quorum sensing, for concentration dependent biosynthesis of the lantibiotic [23]. To ensure that the producer organism is not affected by its own lantibiotic, immunity proteins LanI, LanF, LanE and LanG are also present which provide self-resistance. LanI is a lipoprotein and LanFEG are ABC transporters, which pump the lantibiotic outside, thus, preventing from self-damage [24], [25].

Type IB lantibiotic cluster can further be either a single or two-component lantibiotic encoding cluster. Single component lantibiotics like mersacidin [26] and lacticin 481 [8] have a single antimicrobial peptide. The two-component lantibiotics like haloduracin [27], [28] and lichenicidin [29], [30] consist of two peptides designated as α and β peptides, which show antimicrobial activity through a synergistic effect. The two peptides are highly antimicrobial together but show little or no activity separately. In case of two-component lantibiotic, where the two precursor peptides show high sequence identity, like in case of cytolysin (i.e. 43%) from *Enterococcus faecalis* [31], there is a single LanM to process both the precursor peptides (**Table 1**). In examples where the precursor peptides show low sequence identity, like 17% in case of lichenicidin [29] and 12% in haloduracin [27], two separate LanM enzymes process the respective precursor peptides. Some unique clusters have also been identified which contain more than two precursor peptides for a single LanM. For, example, upto 29 precursor peptides (with 37–84% sequence identity) have been identified in *Prochlorococcus marinus* MIT9313, of which 17 were experimentally found to be processed by a single LanM only [32], showing the highly promiscuous nature of the LanM.

**Table 1:** Sequence identity among the precursor peptides vs. the number of LanM.

Lantibiotic/lantibiotic producer identified by genome mining	No. of precursor peptides/types	% identity Among precursor peptides	Number of LanM.	Reference
*Synechocystis* sp. PCC 7509	9/9	6	2	This work
*Streptomyces hygroscopicus* ATCC 53653	2/2	7	2	This work
*Ruminococcus flavefaciens* FD-1	12/7	7–99	2	This work
*Bacillus* sp. 7_6_55CFAA_CT2	3/3	9–21	2	This work
BhtA	2/2	10	2	[51]
Smb	2/2	10	2	[52]
Lacticin 3147	2/2	10	2	[53]
*Bacillus cereus* FRI-35	8/4	11–68	2	This work
Haloduracin	2/2	12	2	[27]
Staphylococcin C55	2/2	13	2	[54]
*Streptococcus pneumoniae* SP23-BS72	2/2	14	2	[30]
Lichenicidin	2/2	17	2	[30]
*Chamaesiphon minutus* PCC6605	3/3	19–80	2	This work
*Streptomyces viridochromogenes* DSM 40736	2/2	17	2	This work
*Streptomyces bingchenggensis* BCW-1	2/2	20	2	This work
ProcAs	29/29	37–84	1	[32]
*Streptomyces roseosporus* NRRL 11379	2/2	37	1	This work
*Bacillus cereus* VD 166	3/3	41–51	1	This work
Cytolysin	2/2	43	1	[31]Cox
*Herpetosiphon aurantiacus* ATCC 23779	6/6	47–84	1	[30]
*Ktedonobacter racemifer* DSM 44963	6/6	48–86	1	This work
*Bacillus cereus* SJ1	3/2	80	1	This work

A comparison of the sequence identity of the precursor peptides identified in this study, including those of the well-known lantibiotics, bhtA, smb, lacticin 3147, haloduracin, staphylococcin C55, lichenicidin, procAs and cytolysin with the number of LanM processing enzymes required. Upto ∼20% sequence identity among the precursor peptides, two LanMs are required and 37% and above, a single LanM is sufficient for the processing.

Here, we adopted a strategy of using HalT transporter as a query sequence to identify novel type IB lantibiotic clusters by microbial genome database mining. The strategy resulted in identification of 54 strains, from the top 72 hits that were encoding novel HalT homologs in the genome. Out of these 54 strains, we could identify 24 novel lantibiotic clusters that were encoding genes for precursor peptides, modification enzyme, quorum sensing, transport and immunity.

## Methods

### Screening of genome database

Lantibiotic clusters were identified using PSI-BLAST, by taking the C39 peptidase domain containing transporter of haloduracin, HalT (Protein ID: NP_241317.1) as the driver sequence against genomic database on NCBI. PSI-BLAST was carried out using the default parameters, and the top 72 hits were taken for analysis.

### Bioinformatic analysis of LanT homolog containing gene clusters

In cases where novel LanT-like genes were identified, the arrangement of adjacent genes was visualized using the genome viewer on NCBI. The individual ORFs obtained were subjected to both, BLAST with known lantibiotic clusters and analysis with the Conserved Domain Database (www.ncbi.nlm.nih.gov/structure/cdd/cdd.shtml), to identify potential genes involved in lantibiotic production, immunity and quorum sensing. LanRK system was also located using Microbial Signal Transduction database (MiST, http://mistdb.com). Immunity and transporter proteins were analysed with TMHMM (http://www.cbs.dtu.dk/services/TMHMM) and CELLO [33] to ensure their membrane association. The selected genomes were also analyzed using BAGEL2 [18], a web based bacteriocin mining tool. In clusters, where most of the genes were found to be present but LanA encoding genes were not annotated, intergenic regions were inspected using ORF finder (www.ncbi.nlm.nih.gov). Properties of the identified precursor peptides (**Table S1**) were analyzed with APD2 [34].

### Phylogenetic analysis

Alpha and beta precursor peptides in a putative two-component lantibiotic cluster were identified by multiple sequence alignment with ClustalW2 and their phylogenetic analysis with known alpha and beta precursor peptides, of well-known lantibiotics. The evolutionary history was inferred using the neighbour-joining method in MEGA5 [35].

## Results

**Figure 1:**
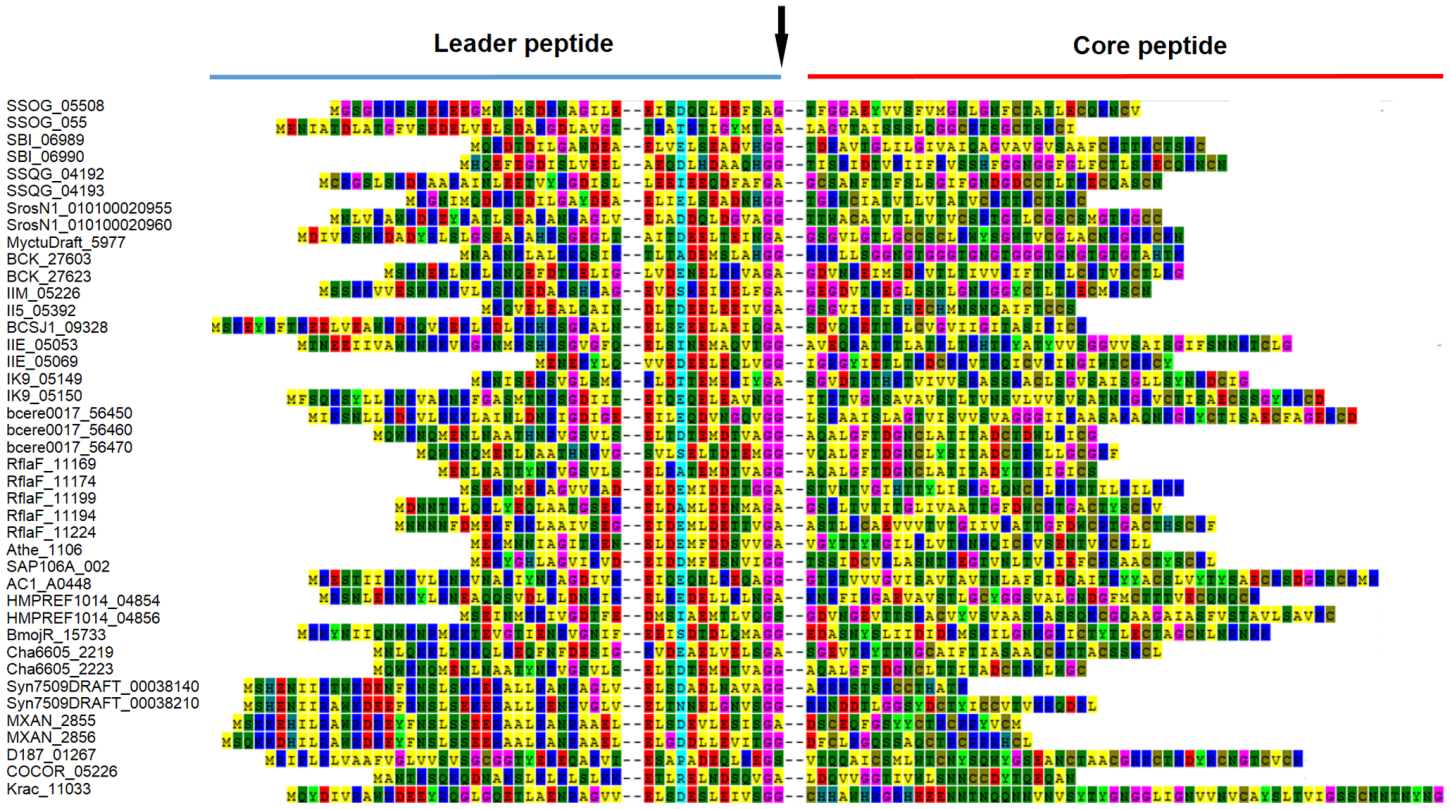
Diverse structures of identified putative lantibiotic precursor peptides. Thirty nine selected lantibiotic precursor peptides shown in ClustalW alignment using MEGA5 [35]. The locus tag is given on the left of the sequence and the amino acid position is given on the right. The cleavage site of the leader peptides is indicated by an arrow. The precursor peptide D187_01267 from *C. fuscus* DSM 2262 has eight cysteine residues, the highest among all the identified precursor peptides.

Using LanT as a mining tool, an *in silico* approach was followed to identify novel type IB lantibiotic clusters in bacterial genome. LanT is a conserved C39 peptidase domain containing lantibiotic transporter and is an integral part of the Type IB clusters i.e. clusters encoding LanM and double glycine motif containing precursor peptide(s). HalT protein of haloduracin biosynthetic cluster [27], [28] was selected as a driver sequence for PSI-BLAST search of the NCBI database. The top 72 hits obtained had a significantly low e-value (<6e-127) including those involved in the production of the lantibiotics like lichenicidin, g_sp_G11MC16, geobacillin, salivaricin (**Table 2**), and some putative lantibiotic clusters [18], [30], [36], thus, proving the effectiveness of our mining approach. Besides the proven ones, 59 novel LanT homologs were identified in 54 bacterial strains (having 34–50% identity with HalT), among the representatives of actinobacteria, firmicutes, cyanobacteria, proteobacteria and chloroflexi, which have not been documented earlier for having any association with the lantibiotic production (**Table 2**). Out of the 54 strains, we could identify 24 novel lantibiotic clusters. In rest of the strains, the identified HalT homolog was either orphan or a complete cluster could not be established. All these 24 clusters are discussed here and the identified lantibiotic precursor peptide sequences are given in detail (**Table S1**) along with their ClustalW alignment (**Fig. 1**). They include 13 clusters having a single LanM and 8 encoding two LanM determinants. Three of the LanT homologs have been found to be associated with LanBC proteins, instead of LanM.

**Table 2:** LanT homologs identified by PSI-BLAST of the NCBI database.

S.No.	Organism	Protein ID	Locus tag*	I	Lantibiotic	Associated protein/modification enzyme
1	*Bacillus halodurans* C-125	NP_241317	BH0451	100	Haloduracin	LanM
2	*Bacillus cereus* FRI-35	YP_006601542.1	BCK_27663	50	Unknown	LanM
3	*Bacillus mycoides* Rock3–17	ZP_04160688	Bmyco0003_57320	50	Unknown	LanM
4	*Bacillus cereus* BAG3X2-1	ZP_17399291.1	IE3_05674	50	Unknown	LanM
5	*Bacillus cereus* ATCC 4342	ZP_04287173.1	bcere0010_52930	50	Unknown	LanM
6	*Bacillus* sp. 7_6_55CFAA_CT2	ZP_09359395.1	HMPREF1014_04858	52	Unknown	LanM
7	*Bacillus licheniformis* DSM 13 = ATCC 14580	YP_081202.1	BL00929	50	Lichenicidin	LanM
8	*Geobacillus* sp. G11MC16	ZP_03149641	G11MC16DRAFT_3400	47	G_sp_G11MC16	LanM
9.	*Geobacillus thermodenitrificans* NG80-2	YP_001126158.1	GTNG_2061	47	Geobacillin	LanM
10.	*Bacillus* cereus Rock 1–3	ZP_04248716.1	bcere0017_56490	46	Unknown	LanM
11.	*Bacillus mojavensis* RO-H-1	ZP_10514325.1	BmojR_010100015748	46	Unknown	LanM
12.	*Bacillus cereus* VD166	ZP_17620816.1	IK9_05143	45	Unknown	LanM
13.	*Staphylococcus epidermidis* plasmid SAP106A	YP_006939047.1	SAP106A_001	41	Unknown	LanM
14.	*Caldicellulosiruptor bescii* DSM 6725	YP_002572986.1	Athe_1112	42	Unknown	LanM
15.	*Bacillus thuringiensis serovar huazhongensis* BGSC 4BD1	ZP_04085253.1	Bthur0011_29240	42	Unknown	LanM
16.	*Bacillus cereus* VD107	ZP_17590370.1	IIM_05224	39	Unknown	LanM
17.	*Clostridium cellulovorans* 743B	YP_003845652.1	Clocel_4227	39	[18]	LanM
18	*Streptococcus salivarius* M18	ZP_12658203.1	SSALIVM18_09011	39	Salivaricin 9	LanM
19	*Ruminococcus flavefaciens* FD-1	ZP_06143780.1	RflaF_010100011229	38	Unknown	LanM
20	*Bacillus cereus* MSX-A1	ZP_17542274.1	II5_05402	38	Unknown	LanM
21	*Bacillus cereus* BAG4X12-1	ZP_17415509.1	IE9_04709	38	Unknown	LanM
22	*Bacillus cereus* VD102	ZP_17584421.1	IIK_05109	39	Unknown	LanM
23	*Bacillus cereus* Q1	YP_002532641.1	BCQ_4951	39	Cereicidin	LanM
24	*Streptococcus pneumoniae* SPNA45	YP_006701771.1	SPNA45_01356	39	Unknown	LanC
25	*Streptococcus pneumoniae* SP23-BS72	ZP_01834980.1	CGSSp23BS72_03643	39	[30]	LanM
26	*Streptococcus pneumoniae* 2070035	ZP_15668142.1	AMCSP03_001176	39	Unknown	LanM
27	*Bacillus cereus* VPC1401	YP_004050050.1	pLVP1401_35	38	Unknown	LanM
28	*Bacillus cereus* SJ1	ZP_07055840.1	BCSJ1_09338	38	Unknown	LanM
29	*Nitrolancetus hollandicus* Lb	ZP_10246169.1	NITHO_5020003	38	Unknown	LanM
30	*Herpetosiphon aurantiacus* DSM 785	YP_001544640.1	Haur_1869	38	Unknown	LanM
31	*Cystobacter fuscus* DSM 2262	ZP_21238522.1	D187_01268	36	Unknown	LanB, LanC, LanO
32	*Streptococcus mitis* SK616	ZP_13519814.1	HMPREF1045_1736	38	Unknown	LanM
33	*Herpetosiphon aurantiacus* DSM 785	YP_001546505.1	Haur_3741	38	Unknown	DUF4135^a^
34	*Clostridium cellulovorans* 743B	YP_003845649.1	Clocel_4224	35	[18]	LanM
35	*Ktedonobacter racemifer* DSM 44963	ZP_06966373.1	Krac_11031	37	Unknown	LanM
36	*Streptomyces roseosporus* NRRL 15998	ZP_06586195.1	SSGG_04023	37	Unknown	LanM
37	*Streptomyces roseosporus* NRRL 11379	ZP_04710453.1	SrosN1_010100020975	37	Unknown	LanM
38	*Paenibacillus polymyxa* SC2	YP_003948757.1	PPSC2_c4567	39	Paenibacillin	LanM
39	*Synechocystis* sp. PCC 7509	ZP_21039750.1	Syn7509DRAFT_00038090	38	Unknown	LanM
40	*Streptococcus* sp. M334	ZP_08052596.1	HMPREF0851_01906	37	Unknown	LanM
41	*Ktedonobacter racemifer* DSM 44963	ZP_06967182.1	Krac_11909	37	Unknown	None
42	*Cystobacter fuscus* DSM 2262	ZP_21237264.1	D187_09830	37	Unknown	HylD
43	*Chondromyces apiculatus* DSM 436	ZP_11024177.1	A176_0298	36	Unknown	LanM
44	*Myxococcus xanthus* DK 1622	YP_631064.1	MXAN_2853	36	Unknown	LanM
45	*Haliangium ochraceum* DSM 14365	YP_003270208.1	Hoch_5839	36	Unknown	HylD
46	*Chamaesiphon minutus* PCC 6605	YP_007096826.1	YP_007096826.1	38	Unknown	LanM
47	*Bacillus amyloliquefaciens* subsp. *plantarum* YAU B9601-Y2	YP_005423188.1	BANAU_3852	36	Unknown	LanM
48	*Bacillus* sp. HIL-Y85/54728	CAB60262.1	mrsT	36	Mersacidin	LanM
49	*Bacillus* sp. 5B6	ZP_10044966.1	MY7_3676	36	Unknown	LanM
50	*Plesiocystis pacifica* SIR-1	ZP_01907120.1	PPSIR1_22636	35	Unknown	None
51	*Cystobacter fuscus* DSM 2262	ZP_21231682.1	D187_03026	36	Unknown	HylD
52	*Myxococcus fulvus* HW-1	YP_004667385.1	LILAB_22040	36	Unknown	LanM
53	*Bacillus cereus* HuB4-10	ZP_17519727.1	IGK_05428	34	Unknown	LanM
54	*Chondromyces apiculatus* DSM 436	ZP_11024563.1	A176_0694	36	Unknown	LanM
55	*Corallococcus coralloides* DSM 2259	YP_005371187.1	COCOR_05231	35	Unknown	LanB, LanC, LanO
56	*Stigmatella aurantiaca* DW4/3-1	ZP_01462269.1	STIAU_4626	36	[30]	HylD
57	*Streptomyces hygroscopicus* ATCC 53653	ZP_07297428.1	SSOG_05511	35	Unknown	LanM
58	*Bacillus cereus* F65185	ZP_04206898.1	bcere0025_58960	35	Unknown	LanM
59	*Bacillus cereus* VD045	ZP_17565749.1	IIE_05074	35	Unknown	LanM
60	*Streptococcus pneumoniae* 2080913	ZP_15697159.1	AMCSP17_001014	38	Unknown	LanC
61	*Streptococcus pneumoniae* 2061617	ZP_15690681.1	AMCSP02_001061	38	Unknown	None
62	*Streptococcus pneumoniae* GA41277	ZP_12810048.1	SPAR67_1039	38	Unknown	None
63	*Bacillus cereus* VD156	ZP_17615132.1	IK7_05888	35	Unknown	LanM
64	*Streptomyces viridochromogenes* DSM 40736	ZP_07305308.1	SSQG_04195	34	Unknown	LanM
65	*Anaeromyxobacter* sp. Fw109-5	YP_001380302.1	Anae109_3122	34	Unknown	HylD
66	*Clostridium perfringens* B str. ATCC 3626	ZP_02636759.1	AC1_A0446	33	[30]	LanM
67	*Mycobacterium tusciae* JS617	ZP_09685921.1	MyctuDRAFT_5975	32	Unknown	LanB
68	*Microcystis aeruginosa* NIES-843	YP_001658599.1	MAE_35850	35	Unknown	HylD
69	*Microcystis aeruginosa* PCC 9717	ZP_18819417.1	MICAB_840008	36	Unknown	HylD
70	*Acaryochloris* sp. CCMEE 5410	ZP_09249140.1	ACCM5_010100017748	34	Unknown	HylD
71	*Microcystis aeruginosa* PCC 9808	ZP_18838817.1	MICAG_730010	35	Unknown	LanM
72	*Streptomyces bingchenggensis* BCW-1	YP_004965238.1	SBI_06987	34	Unknown	LanM

*as on 15 February 2013.

a DUF4135 – N-terminal conserved domain of LanM proteins.

I- Percentage identity with HalT.

### Lantibiotic gene clusters identified in actinobacteria

#### Identification of novel *Streptomyces* associated lantibiotic gene clusters

**Figure 2:**
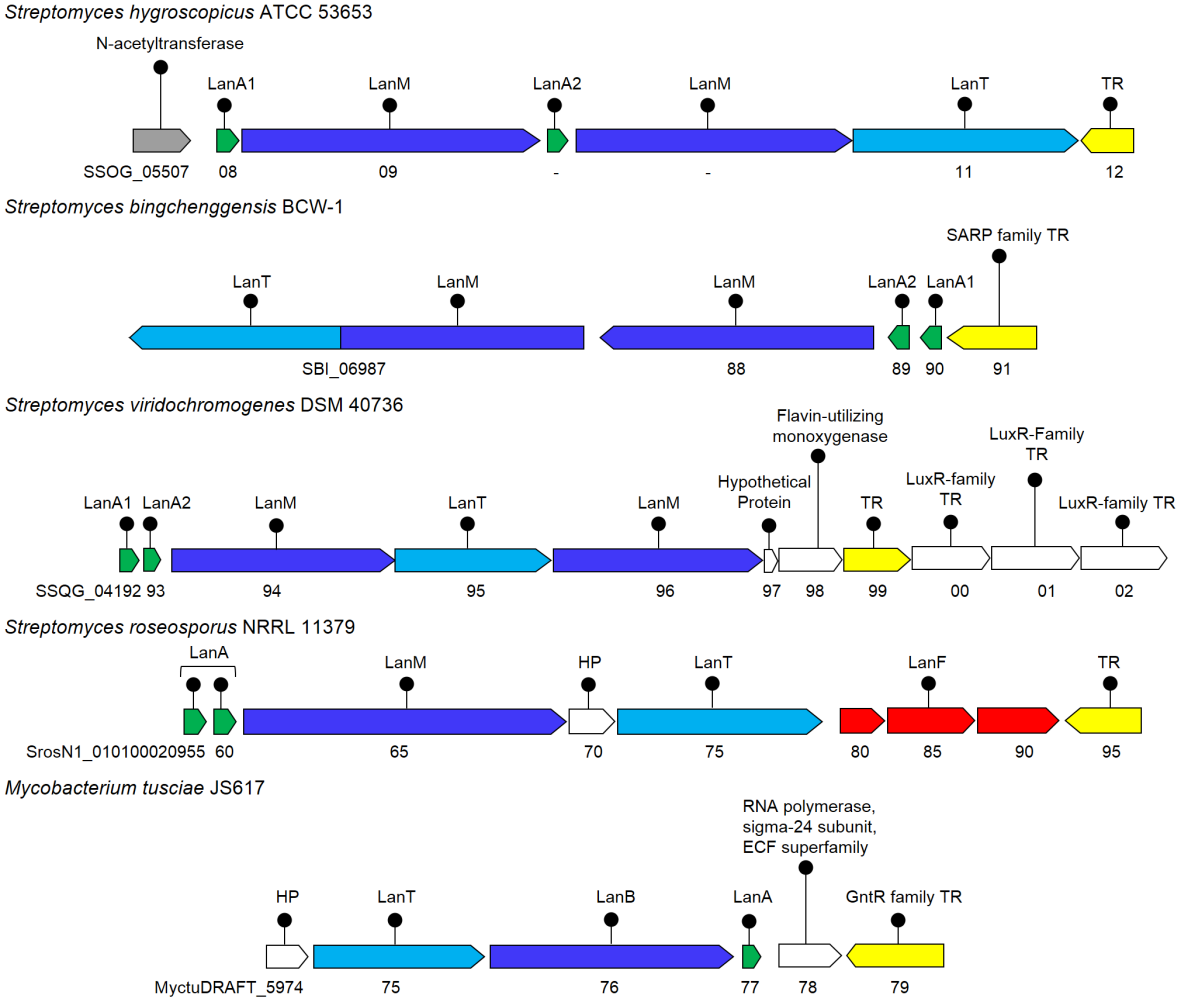
Cluster organization of the putative lantibiotic biosynthetic genes identified in actinobacteria, *Streptomyces* and *Mycobacterium*. *S. hygroscopicus* ATCC 53653, *S. bingchenggensis* BCW-1, *S. viridochromogenes* DSM 40736 are shown here, encoding two LanM determinants and the phylogenetic analysis (**Fig. 3**) of the precursor peptides suggested that these clusters might encode a putative two-component lantibiotic. LanA1 and LanA2 represents alpha and beta precursors. In *Mycobacterium tusciae*, the double glycine motif containing precursor peptide was found to be encoded with the LanB determinant, enzyme for dehydration of type IA precursor peptides. HP - Hypothetical Protein; TR - Transcriptional Regulator; immunity genes which cannot be assigned as LanF/E/G are shown in red color.

*Streptomyces* are Gram-positive bacteria with a unique capacity for the production of a multitude of varied and complex secondary metabolites. A number of lantibiotics associated with *Streptomyces* have been identified previously, like type IB lantibiotics ancovenin [37] and duramycin C [38]. We have identified novel clusters, both with single and two LanM determinants, in four representatives of *Streptomyces* (**Fig. 2**), which are discussed here.

#### *Streptomyces roseosporus* NRRL 11379

*S. roseosporus* produces the antibiotic daptomycin, which is effective against vancomycin and methicillin-resistant *Staphylococcus aureus*. The draft genome sequence of *S. roseosporus*, revealed a cluster (**Fig. 2**) with two putative precursor peptides (SrosN1_010100020955 and 60), a single LanM determinant (SrosN1_010100020965, 24% identical to HalM of haloduracin) and putative immunity genes (SrosN1_010100020980, 85 and 90). The precursor peptides had 37% identity, which was intermediate between haloduracin (22%) and cytolysin precursor peptides (∼43%), that are processed by two and single LanM, respectively (**Table 1**). No other LanM encoding gene was present in the whole genome. In order to have an idea of the relation between the percent identity among the precursor peptides and the number of LanMs that can process them, the putative precursor peptides identified in this biosynthetic cluster were compared with those of the well-known lantibiotics, for their identity score (**Table 1**). The analysis indicated that, probably the precursor peptides are processed by a single LanM. In case of prochlorococcins also, a single LanM has been shown to process 17 out of 29 proposed precursor peptides, having 37–84% identity [32]. Therefore, it can be hypothesized that with an intermediate identity of 37%, they would be processed by a single LanM, thus, marking a probable threshold value in the identity of precursor peptides, in light of the examples of experimentally proven lanthipeptides.

#### *Streptomyces hygroscopicus* ATCC 53653

**Figure 3:**
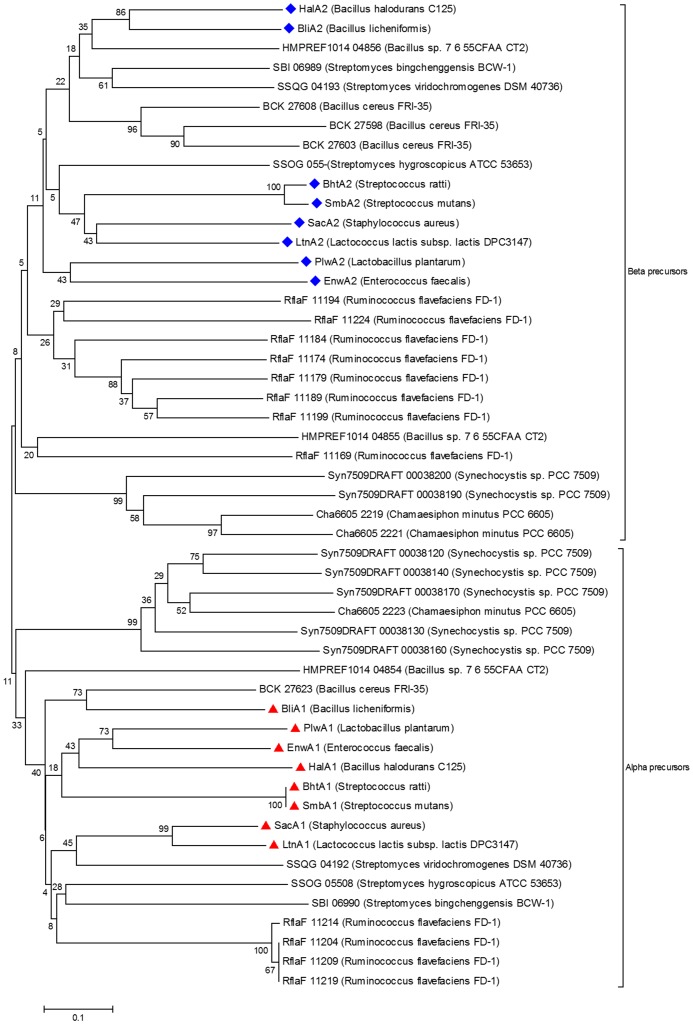
Phylogenetic analysis of the identified precursor peptides with α and β precursors of well-known two-component lantibiotics. Alpha and beta precursor peptides of the two-component lantibiotics smb (SmbA1: BAD72777; SmbA2:BAD72776), bhtA (BhtA1:AAZ76603; BhtA2:AAZ76602), lichenicidin (LchA1:ADM36018; LchA2:ADM36017), lacticin 3147 (LtnA1:O87236; LtnA2:O87237), staphylococcin C55 (SacA1:BAB78438; SacA2:BAB78439), haloduracin (HalA1:BAB04173; HalA2:BAB04172), plantaricin W (PlwA1:AAG02567; PlwA2:AAG02566), enterocin W (EnwA1:BAL50001; EnwA2:BAL50002.1) and lichenicidin (BliA1:Q65DC4.1; BliA2:P86720.1) were taken for the phylogenetic analysis. Alpha precursor peptides are marked with a pyramid and beta precursor peptides with a diamond. The percentage of replicate trees in which the associated taxa clustered together in the bootstrap test (500 replicates) are shown next to the branches. The tree is drawn to scale, with branch lengths in the same units as those of the evolutionary distances used to infer the phylogenetic tree. The evolutionary distances were computed using the p-distance method and are in the units of the number of amino acid differences per site.

*S. hygroscopicus*, also known as *S. himastatinicus* ATCC 53653 is a soil isolate from Himachal Pradesh, India and produces the antitumor agent himastatin. It is an aerobic mesophilic terrestrial bacterium. The genomic annotation of *S. hygroscopicus* showed the LanM determinant (SSOG_05509, 22% identical to HalM) as conserved hypothetical protein, LanT determinant (SSOG_05511) as the lantibiotic transporter, and two putative precursor peptides (SSOG_05508 and 10) as the hypothetical proteins. Since, the sequence identity among these putative precursor peptides was very low i.e. 7% (**Table 1**), therefore we anticipated that it should have a second LanM also. When the same region was analysed with ORF finder (NCBI), we could identify a second LanM determinant (23% identical to HalM; **Fig. 2**) with a rather shorter precursor peptide encoding ORF than SSOG_05510, adjoining the LanT homolog. Phylogenetic analysis of both the putative precursor peptides (**Fig. 3**) showed that these are homologs of the precursor peptides of two-component lantibiotics. Both of these features led us to conclude that the identified cluster might encode for a two-component lantibiotic.

#### *Streptomyces bingchenggensis* BCW-1

**Figure 4:**
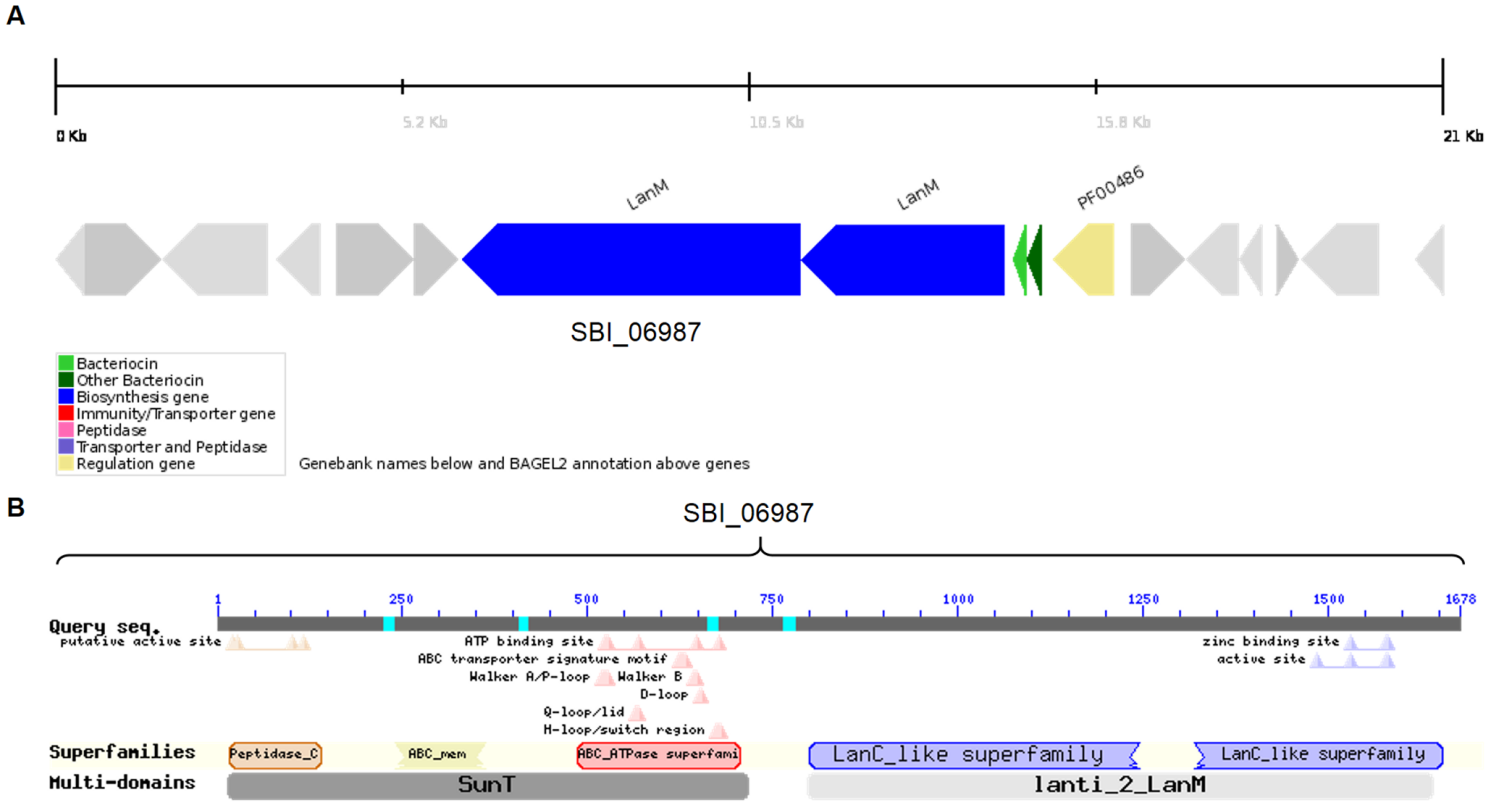
Comparison of BAGEL2 and CDD results for the ORF SBI_06987 encoded in the *Streptomyces bingchenggensis* BCW-1 genome. (A) BAGEL2 showing SBI_06987 as LanM only, while the hidden transporter could not be identified by the software. (B) CDD showing the conserved domain for LanM (LanC like superfamily) as well as C39 protease domain (peptidase_C, ATPase domain and ABC_membrane domain) containing transporter, LanT.

*S. bingchenggensis* is a soil bacterium isolated from Harbin, China. It produces milbemycins (commercially important anthelmintic macrolide compounds), other insecticidal antibiotics, and a few cyclic pentapeptides and is thus interesting for its secondary metabolite producing capabilities [39]. Our genome analysis of *S. bingchenggensis* resulted in the identification of a cluster (**Fig. 2**) consisting of a LanM determinant (SBI_06988; 18% identical to HalM) and two putative precursor peptides (SBI_06989 and 90), that were originally annotated as hypothetical proteins, and a LanT homolog (SBI_06987) of ∼1600 amino acid residues, annotated as cytolysin B transport protein. The size of LanT appeared large enough, for it to be just a transport protein (**Table 3**). Also the precursor peptides were found to have an identity of 20% among themselves, which being very low (**Table 1**), suggested that the cluster should have two LanMs [27]. Phylogenetic analysis (**Fig. 3**) also suggested that this cluster might encode for a two-component lantibiotic cluster. Using CDD, we could identify a second LanM, fused with the C39 protease and the ABC transporter domain, in this large protein sequence (**Fig. 4**). Though the cluster was also analyzed by BAGEL2, it predicted this single ORF as LanM and could not identify the hidden transporter. It will be interesting to know with wet lab experiments, if this tri-domain protein is operative in the formation of an active lantibiotic. When this long putative transporter sequence was subjected to BLAST, no other similar example could be identified on NCBI Entrez database. The second LanM could only be identified by using a consolidation of BLAST, CDD and BAGEL2, as it was fused with the LanT.

**Table 3:** Approximate size of the lantibiotic biosynthetic proteins.

Lantibiotic cluster protein	Approximate length (Amino acids)
LanA	50–100
LanB	600–1000
LanC	∼400
LanT (ABC Transporter)	∼600
LanT (ABC Transporter+ C39 Peptidase)	∼700
LanT*(ABC transporter+ C39 Peptidase+LanM)	∼1600
LanM	900–1000
LanFGE	200–300
LanR	200–250
LanK	400–600

*SBI_06987 identified in *Streptomyces bingchenggensis* BCW-1.

Approximate size of the proteins encoded in a type I lantibiotic biosynthetic cluster along with that of the identified ORF SBI_06987 (Protein ID: YP_004965239.1), in *Streptomyces bingchenggensis* BCW-1.

#### *Streptomyces viridochromogenes* DSM 40736

*S. viridochromogenes* is a producer of avilamycin A, an oligosaccharide antibiotic. Its genome analysis revealed a lantibiotic cluster (**Fig. 2**) with two putative precursor peptides (SSQG_04192 and 93) and two LanM determinants (SSQG_04194 and 96, showing 18% and 17% identity with HalM), besides the LanT homolog (SSQG_04195). The two LanM genes were found to be flanking the transporter. The cluster might encode for a two-component lantibiotic, as suggested by the phylogenetic analysis of the precursor peptides (**Fig. 3**). Downstream to the cluster, many transcriptional regulators (TR) were present next to the hypothetical protein (SSQG_04197) and the flavin utilizing monoxygenase gene (SSQG_04198).

#### *Mycobacterium tusciae* JS617

*M. tusciae* is a slow growing scotochromogenic mycobacterium that has been found in tapwater. This organism was isolated from the lymph node of an immunocompromised patient and from the respiratory sample of cystic fibrosis patient. Unexpectedly, the cluster identified in *M. tusciae* turned out to be a type IA cluster instead of a type IB cluster (**Fig. 2**). It had a LanT homolog (MyctuDRAFT_5975), a LanB determinant (MyctuDRAFT_5976) and a putative LanA precursor peptide (MyctuDRAFT_5977). The core-peptide region of the identified precursor peptide had a high amount of glycines (∼43%) with serine and threonine residues, but no cysteine at all. Interestingly, the LanC cyclase, which links the cysteine to Dha and Dhb residues for cyclization, was neither found anywhere near the cluster and nor in the bacterial genome.

### Lantibiotic gene clusters identified in firmicutes

#### Identification of novel *Bacillus cereus* associated lantibiotic clusters

*Bacillus* species are rod-shaped, endospore-forming aerobic or facultatively anaerobic, Gram-positive bacteria that are ubiquitous in nature. The many species of the genus exhibit a wide range of physiologic abilities that allow them to live in every natural environment. Many lantibiotics have been discovered from the phyla including lichenicidin, haloduracin, amylolysin etc.

##### *Bacillus cereus* FRI-35 plasmid p03

**Figure 5:**
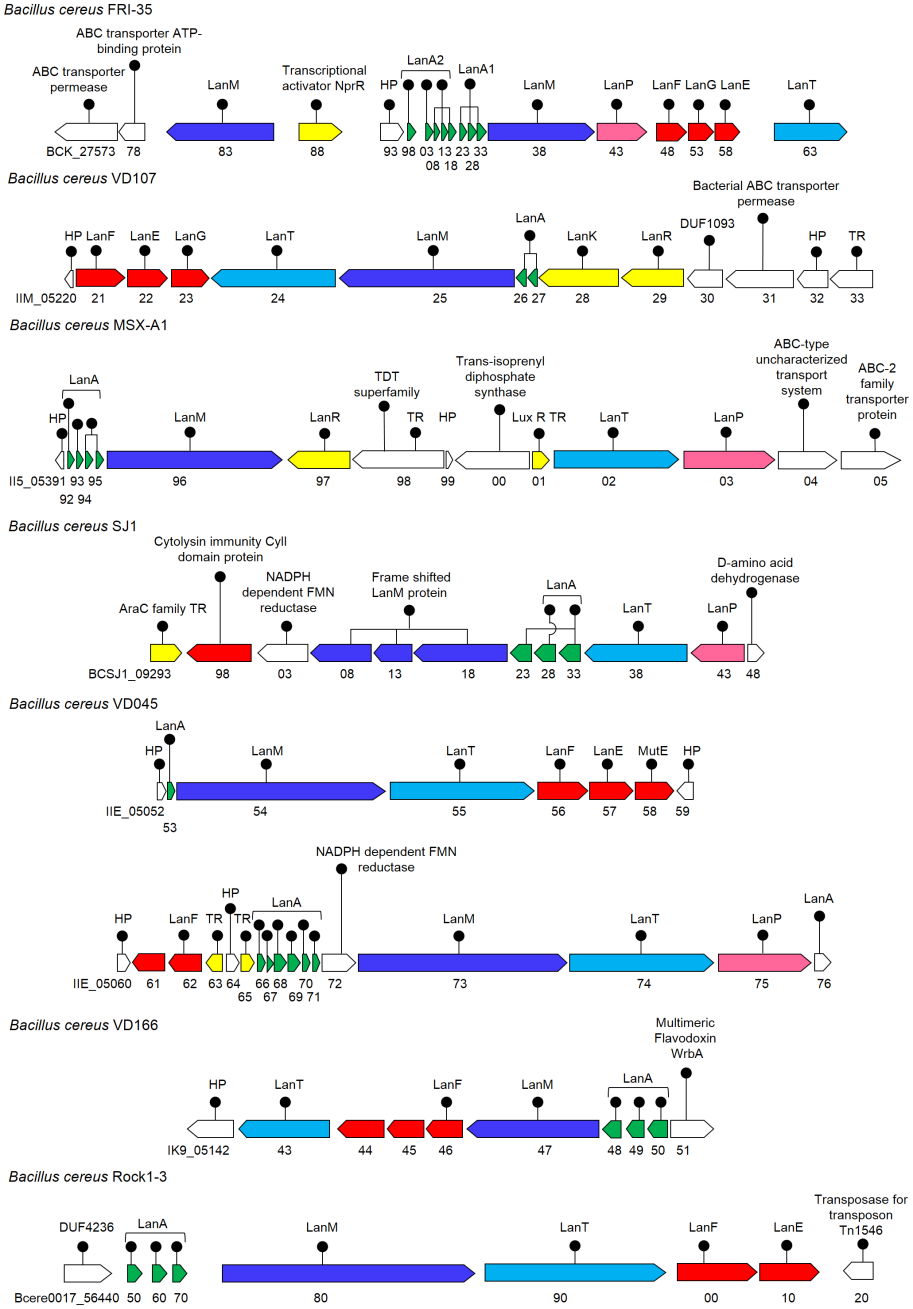
Cluster organization of the putative lantibiotic biosynthetic genes identified in the strains of *Bacillus cereus*. Cluster organization representing a diversity among subspecies of *B. cereus*, which belongs to *Firmicutes*. Only the strain, *B.cereus* FRI-35 was found to encode a putative two-component lantibiotic cluster (**Fig. 3**). Annotation with connected lines represent identical precursor peptides.

Genome analysis of FRI-35 revealed the presence of a two-component lantibiotic cluster (**Fig. 5**) with two LanM determinants (BCK_27583 and BCK_27638, 33% and 25% identical to HalM, respectively) and as many as eight putative double glycine motif containing precursor peptides, with 11–68% identity among them (**Table 1**). The whole biosynthetic cluster was present on the plasmid p03 (Sequence: NC_018499.1), of its four plasmids. Eight precursor peptide encoding genes were identified, which included 3 identical copies each of α (BCK_27608, 13 and 18) and β peptides (BCK_27623, 28 and 33) and two more peptides (BCK_27598 and 03), that fall within the β peptides in the phylogenetic analysis (**Fig. 3**). All the three immunity proteins, LanF (BCK_27648), LanE (BCK_27653) and LanG (BCK_27658) with the LanT homolog (BCK_27663) and an additional LanP, S8 peptidase were also present in the cluster (BCK_27643).

##### *Bacillus cereus* VD107

Genome analysis of VD107 revealed a complete cluster (**Fig. 5**) containing two identical precursor peptides (IIM_05226 and IIM_05227), all the immunity protein determinants, LanE (IIM_05222), LanF (IIM_05221) and LanG (IIM_05223), a LanM protein (IIM_05225, 31% identical to HalM) besides the LanT homolog (IIM_05224). Two-component response regulator elements, LanR (IIM_05229) and LanK (IIM_05228) were also located in the cluster. The putative precursor peptide had 84% similarity with variacin, a lantibiotic produced in an entirely different phyla, actinobacteria.

##### *Bacillus cereus* MSX-A1

Genome analysis of MSX-A1 led to the identification of a lantibiotic cluster (**Fig. 5**) having four almost identical precursor peptides (80–95% identity; II5_05392, 93, 94 and 95), a LanM determinant (II5_05396, having 27% identity with HalM), a LanT homolog (II5_05402) and a LanP (II5_05403). A LanR determinant (II5_05397) of two-component response regulatory system was also present. The cluster was intermediated by an unrelated gene annotated as hypothetical protein, the trans-isoprenyl diphosphate synthase (II5_05400). Two putative ABC transporter genes (II5_05404 and 05) were also found downstream to the cluster next to LanP, which might have a role in immunity.

##### *Bacillus cereus* SJ1

Lantibiotic biosynthetic cluster in SJ1 (**Fig. 5**) was found to contain an apparently frameshifted LanM determinant (BCSJ1_09308, 13 and 18, showing 19%, 28% and 22% identity with HalM), alongwith three putative precursor peptides (BCSJ1_09323, 28, and 33), out of which only BCSJ1_09328 contain a cysteine residue and the other two, although had a double glycine motif, but were devoid of any cysteine (**Table 1**). All these three putative precursor peptides had an identical leader sequence, with variation in the core-peptide region only. Similar to MSX-A1, there was an additional LanP determinant (BCSJ1_09343) present in the cluster, near the LanT homolog (BCSJ1_09338). A cytolysin immunity protein (CylI; BCSJ1_09298) was also found to be present in the cluster.

##### *Bacillus cereus* VD045

Bioinformatic analysis of the draft sequence of VD045 revealed the presence of two putative lantibiotic biosynthetic clusters (**Fig. 5**), present in the vicinity, on the three contigs (conti1.34, 1.35 and 1.36), with their dedicated transporters and immunity genes. First cluster had a single putative precursor peptide (IIE_05053), a LanM (IIE_05054, 19% identical to HalM) and a LanT determinant (IIE_05055), with the putative immunity proteins LanF (IIE_05056), LanE (IIE_05057) and MutE homolog (IIE_05058). In the second cluster, there were six putative precursor peptides (IIE_05066, 67, 68, 69, 70 and 71) with almost identical leader sequences (74–97% identity), a LanM (IIE_05073, 24% identical to HalM), a LanT (IIE_05074) and a LanP (IIE_05075) determinant. An intermediating NADPH dependent FMN reductase protein was also present in the this cluster.

##### *Bacillus cereus* VD166

VD166 genome was found encoding a lantibiotic cluster (**Fig. 5**) comprising of three putative precursor peptides (IK9_05148, 49 and 50), a LanM determinant (IK9_05147, 24% identical to HalM) and a LanT homolog (IK9_05143). Using BAGEL2, only a single putative immunity protein, LanF (IK9_05146) could be identified near the cluster. Since, the immunity genes are normally clustered together, therefore, when the two nearby ORFs (IK9_05144 and 45) were analyzed by TMHMM, CELLO and CDD, they turned out to be transmembrane proteins. Also, the size of these proteins was in the range of the immunity proteins (**Table 3**) which led us to speculate that these ORFs might be encoding for the two immunity proteins, LanG and LanE.

##### *Bacillus cereus* Rock1–3

Genome analysis of Rock1–3 revealed a cluster (**Fig. 5**) with three putative precursor peptides (bcere0017_56450, 60 and 70) besides the LanM (bcere0017_56480, 24% identical to HalM) and LanT determinants (bcere0017_56490) with only two putative immunity proteins, LanF (bcere0017_56500) and LanE (bcere0017_56510). A transposase protein (bcere0017_56520) was also present just downstream of the cluster.

#### Identification of novel lantibiotic clusters in other firmicutes

##### *Ruminococcus flavefaciens* FD-1

**Figure 6:**
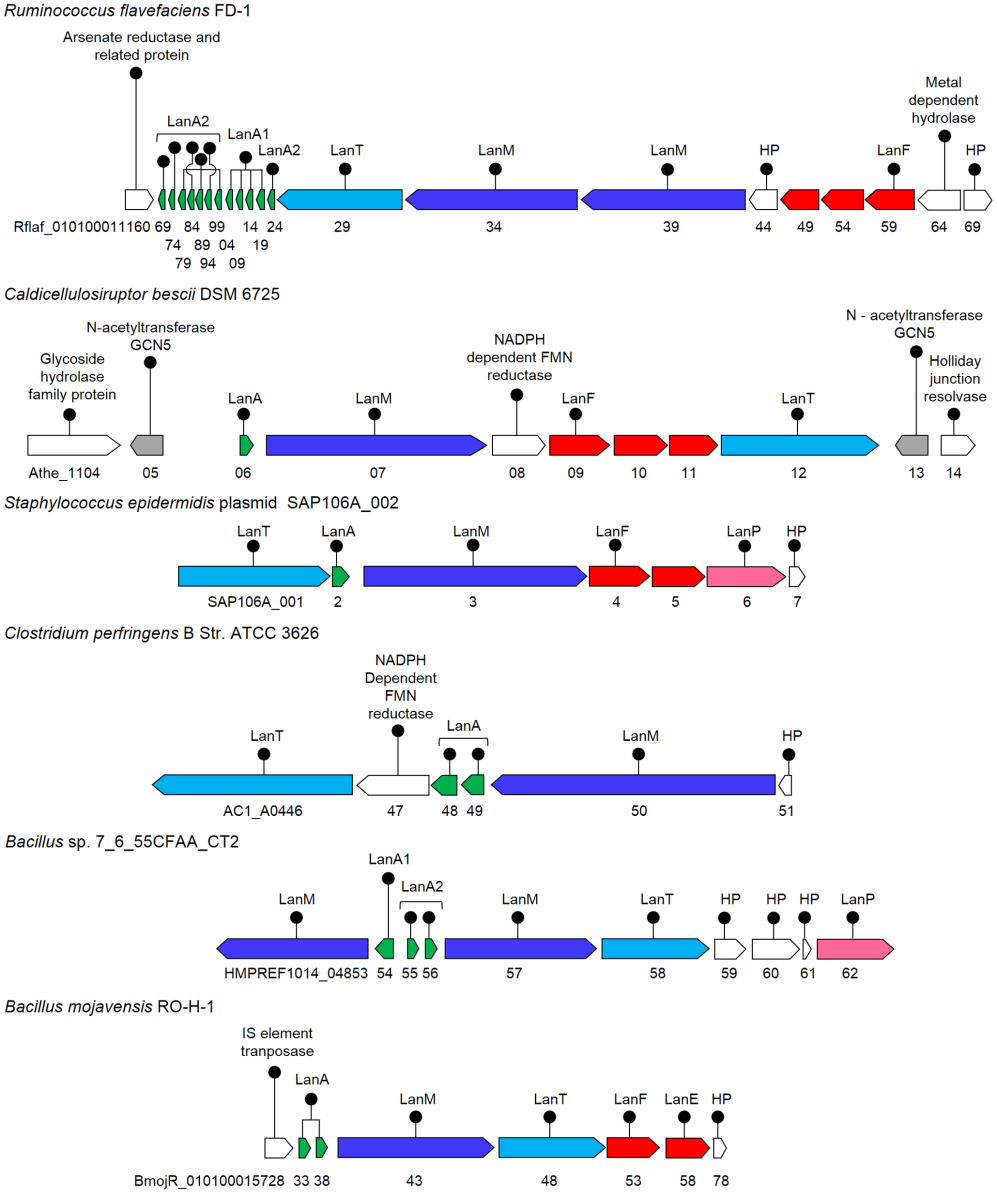
Cluster organization of the putative lantibiotic biosynthetic genes identified in firmicutes other than *B. cereus*. Lantibiotic clusters identified in other *Firmicutes* besides the strains of *Bacillus cereus*. The clusters identified in *R. flavefaciens* FD-1 and *Bacillus* sp. 7_6_55CFAA_CT2 encode two LanM determinants and phylogenetic analysis of the identified precursor peptides suggested that these clusters might encode for a two-component lantibiotic (**Fig. 3**).

*R. flavefaciens* is a Gram-positive cocci in the order ‘*Clostridiales*’ of firmicutes, which is a predominant anaerobic cellulolytic rumen bacterium. It was isolated from bolus containing ruminal microorganisms, which improve rumen function in calves. The culture has the largest known number of fibre-degrading enzymes. In *R. flavefaciens* genome, a cluster (**Fig. 6**) with as many as 12 putative α and β precursor peptides (**Fig. 3**) was identified (RflaF_010100011169, 74, 79, 84, 89, 94, 99 and RflaF_010100011204, 09, 14, 19 and 24) with two LanM (RflaF_010100011234 and 39, showing 24% and 25% identity with HalM, respectively) and a LanF determinant (Rflaf_010100011259). Two more immunity proteins (Rflaf_010100011249 and 54) were identified in this cluster, as that were found in *B. cereus* VD166, discussed earlier.

##### *Caldicellulosiruptor bescii* DSM 6725

*C. bescii*, also known as *Anaerocellum thermophilum* DSM 6725 is a thermophilic, cellulolytic, anaerobic bacterium isolated from a hot spring on the Kamchatka peninsula in Russia. It is the most thermophilic cellulose-degrading organism known [40]. Genome analysis of *C. bescii* revealed the presence of a lantibiotic cluster (**Fig. 6**), with a single putative precursor peptide (Athe_1106), a LanM determinant (Athe_1107, having 26% identity with HalM), a LanT homolog (Athe_1112) and a LanF determinant (Athe_1109). Two more ORFs (Athe_1110 and 11) next to the LanF were identified as immunity proteins. Two N-acetyltransferases (Athe_1105 and 13) were also found at immediate upstream and downstream of the cluster. Since, the lantibiotic paenibacillin has been shown to be acetylated by an acetyltransferase [41], these N-acetyltransferases probably might be playing a similar role here.

##### *Staphylococcus epidermidis* plasmid SAP_106A

*S. epidermidis* is a Gram-positive bacterium, belonging to the genus *Staphylococcus*. It is part of the human skin flora (commensal), and is also found in the mucous membranes of animals. In *S. epidermidis* genome, a cluster was identified on plasmid SAP106A (**Fig. 6**), containing a single putative precursor peptide (SAP_106A002), a LanM determinant (SAP_106A003, having 22% identity with HalM) and an additional LanP protein, besides the C39 protease of LanT homolog (SAP_106A001). A LanF determinant (SAP_106A004) and a probable immunity protein encoding gene (SAP_106A005) was also identified in the cluster.

##### *Clostridium perfringens* B str. ATCC 3626

*C. perfringens* is a rod-shaped, motile mesophilic pathogen belonging to firmicutes, which causes dysenteria, enterocolitis, enterotoxemia, food poisoning and gas gangrene. It was isolated from the intestinal contents of a lamb. Its genome analysis revealed a lantibiotic biosynthetic cluster (**Fig. 6**) with two putative precursor peptides (AC1_A0448 and 49), a LanM determinant (AC1_A0450) besides the LanT homolog (AC1_A0446). An atypical NADPH-dependent FMN reductase gene (AC1_A0447), as found in some of the other clusters, discussed elsewhere, was also present.

##### *Bacillus* sp. 7_6_55CFAA_CT2

This culture has been isolated from the healthy gut biopsy tissue from a patient with Crohn's disease. Its genome was found to contain a cluster (**Fig. 6**) with three putative precursor peptides (HMPREF1014_04854, 55 and 56) and two LanM determinants (HMPREF1014_04853 and 57, 26% and 33% identical to HalM, respectively). Phylogenetic analysis suggested that the cluster might encode for a putative two-component lantibiotic (**Fig. 3**). An S8 peptidase, LanP (HMPREF1014_04862) was also found next to the three hypothetical proteins, besides the bifunctional LanT homolog (HMPREF1014_04858).

##### *Bacillus mojavensis* RO-H-1

Analysis of draft genome sequence of *B. mojavensis* revealed a lantibiotic biosynthetic cluster (**Fig. 6**) comprising of two identical putative precursor peptides (BmojR_010100015733 and 38) associated with a single LanM determinant (BmojR_010100015743, 25% identity with HalM). An associated LanT homolog (BmojR_010100015748) and two immunity protein determinants, LanF (BmojR_010100015753) and LanE (BmojR_010100015758) were also located in the cluster.

#### Lantibiotic gene clusters identified in cyanobacteria

##### *Chamaesiphon minutus* PCC 6605

**Figure 7:**
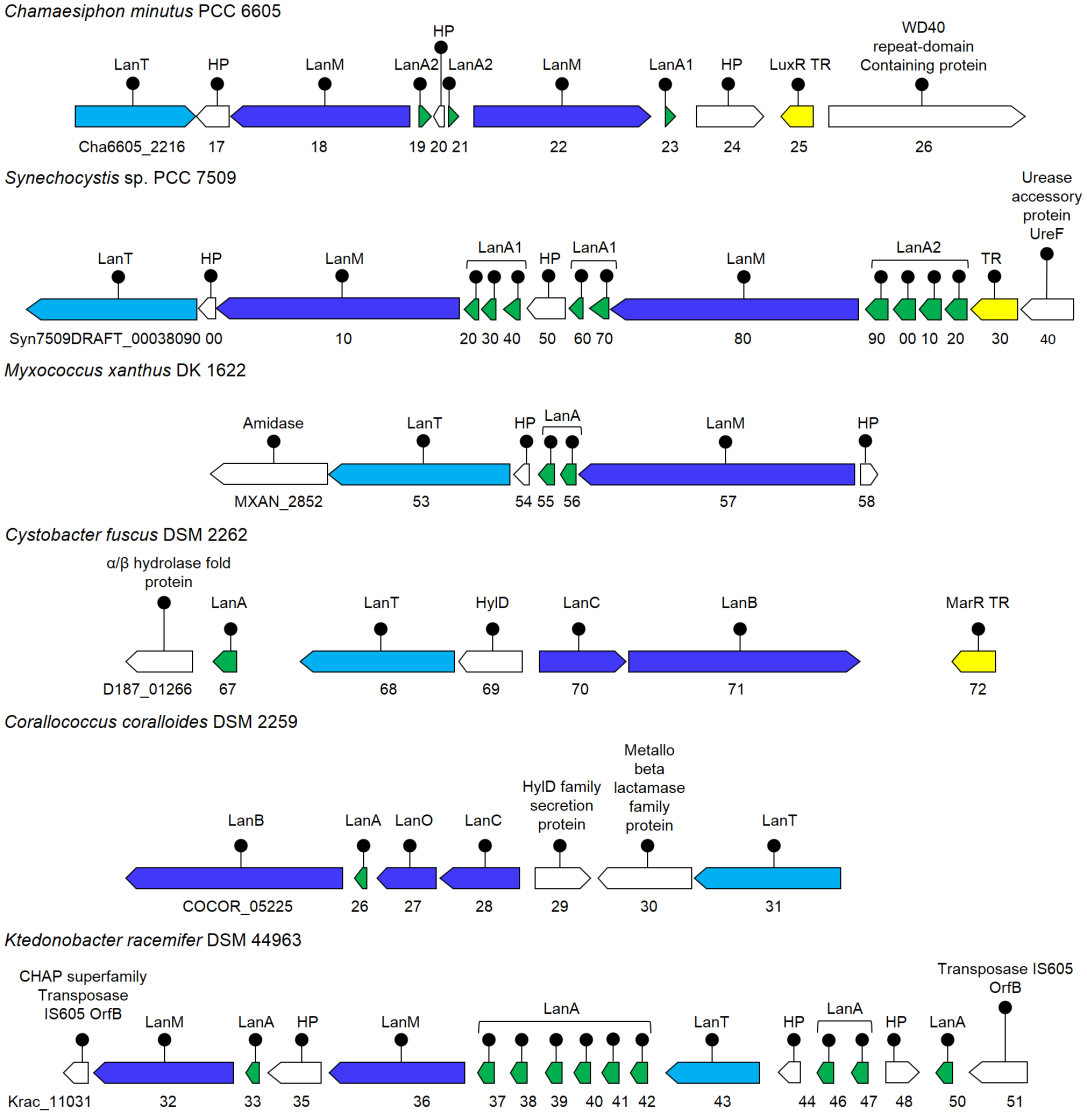
Cluster organization of the putative lantibiotic biosynthetic genes identified in cyanobacteria, proteobacteria and chloroflexi. Putative lantibiotic cluster identified in cyanobacteria: *C. minutus* PCC 6605 and *Synechocystis* sp. PCC 7509; proteobacteria: *M. xanthus* DK 1622, *C. fuscus* DSM 2262, *C. coralloides* DSM 2259; chloroflexi; *K. racemifer* DSM 44963.

*C. minutus* is a free living mesophilic cyanobacteria belonging to order *Chroococcales*, which was isolated from freshwater at Berkeley, USA. Its genome was found to encode a putative two-component lantibiotic cluster (**Fig. 7**) with three putative precursor peptides (Cha6605_2219, 21 and 23) and two LanM determinants (Cha6605_2218 and 22, 29% and 24% identical to HalM), besides the identified LanT homolog (Cha6605_2216).

##### *Synechocystis* sp. PCC 7509

*Synechocystis* sp. is a free living Gram-negative mesophilic cyanobacteria of the order *Chroococcales*, isolated from Switzerland. Analysis of the draft sequence of *Synechocystis* sp. revealed the presence of a lantibiotic cluster (**Fig. 7**) containing as many as nine putative precursor peptides (Syn7509DRAFT_00038120, 130, 140, 160, 170, 190, 200, 210 and 220) with two LanM determinants (Syn7509DRAFT_00038110 and 80, 31% and 23% identical to HalM, respectively) and a LanT homolog (Syn7509DRAFT_00038090). Phylogenetic analysis of the precursor peptides (**Fig. 3**) suggested the cluster to be a putative two-component lantibiotic cluster.

#### Lantibiotic gene clusters identified in proteobacteria

##### *Myxococcus xanthus* DK 1622

*M. xanthus* is an aerobic mesophilic terrestrial proteobacteria. It has a large genome (9.14 Mb) containing more than 1500 gene duplications. These gene duplications more commonly involve genes used in cell to cell signaling, molecule sensing and transcription control. A lantibiotic cluster (**Fig. 7**) with two putative precursor peptides (MXAN_2855 and 56), a LanM determinant (MXAN_2857, having 27% identity with HalM) and the LanT (MXAN_2853) homolog was identified in its genome. Two more peptides, encoded as hypothetical proteins (MXAN_2854 and 58), have been identified in this cluster which though are of the precursor peptide length, do not possess the characteristic features of the lantibiotic precursor peptide.

##### *Cystobacter fuscus* DSM 2262

*C. fuscus* is a Gram-negative aerobic bacteria of the order *Myxococcales*. Similar to *M. tusciae* JS617, its genome analysis also identified a putative type IA lantibiotic cluster (**Fig. 7**) comprising of a putative precursor peptide (D187_01267), a LanB (D187_01271) and a LanC determinant (D187_01270) associated with the LanT homolog (D187_01268). Two more LanT homologs without any associated modification enzymes were also identified elsewhere in the genome (**Table 2**), which might also be involved in processing of the double glycine motif containing precursor peptides. The precursor peptides processed by LanBC proteins generally have a proline at -2 position, but here instead, double glycine motif was present. Noteworthy here is the presence of as many as eight cysteine residues of the total sixteen modifiable residues in the core peptide region (**Fig. 1**), maximum in any lanthipeptide discovered till date. These eight cysteine residues may contribute to form eight lanthionine rings, thus, probably making it more stable.

##### *Corallococcus coralloides* DSM 2259

*C. coralloides* is an aerobic terrestrial mesophilic Gram-negative bacteria of phylum proteobacteria. Genome analysis of *C. coralloides* also revealed the presence of a novel type IA cluster associated with the C39 containing LanT protein (**Fig. 7**). Three modification enzymes, LanB (COCOR_05225), LanC (COCOR_05228) and LanO (COCOR_05227) determinants were present in the cluster, along with the double glycine motif containing precursor peptide (COCOR_05226), as was in *M. tusciae* and *C. fuscus* discussed above.

#### Lantibiotic gene cluster identified in chloroflexi

##### *Ktedonobacter racemifer* DSM 44963

**Figure 8:**
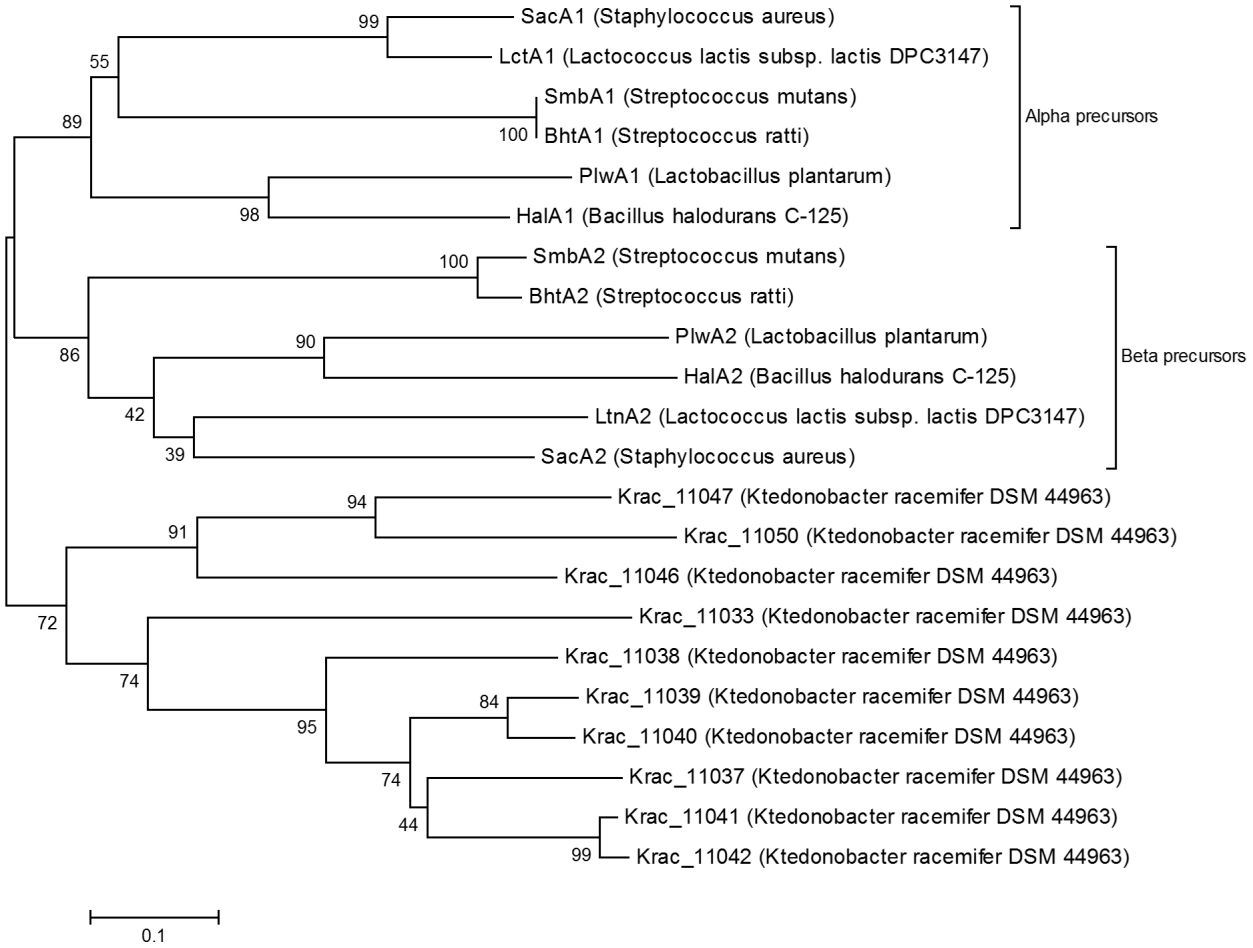
Phylogenetic analysis of the putative precursor peptides identified in *Ktedonobacter racemifer* DSM 44963. The ten identified precursor peptides in *K. racemifer* DSM 44963 formed a separate clade from the alpha and beta precursor peptides of well-known lantibiotics.

*K. racemifer* is an aerobic, filamentous, non-motile, spore-forming Gram-positive heterotroph, which was isolated from a soil sample in Italy and represents a new phylogenetic class *Ktedonobacteria*, though it shares some morphological features with the actinobacteria. This culture is of special interest because it was the first cultivated representative of a deep branching unclassified lineage of otherwise uncultivated environmental phylotypes, tentatively located within the phylum ‘chloroflexi’ [42]. The draft genome sequence of *K. racemifer* revealed the presence of a lantibiotic biosynthetic cluster (**Fig. 7**) with as many as ten putative precursor peptides (Krac_11033, 37, 38, 39, 40, 41, 42, 46, 47 and 50), two LanM determinants (Krac_11032 and 36, showing 30% and 27% identity with HalM) and the LanT homolog (Krac_11043). A second LanT homolog (Krac_11909; not shown) not associated with any lantibiotic precursor peptide was also present in the genome. Since there are two LanMs present in the cluster, so it was expected that the phylogenetic analysis would distribute the putative precursor peptides among the two clades of α and β precursors. Instead, the precursor peptides formed a separate group, unrelated with α and β precursor peptides (**Fig. 8**), suggesting that the cluster is probably not a two-component lantibiotic cluster.

### Discussion

Genome mining is being extensively and successfully done these days using bioinformatic tools, to identify novel genes and gene clusters. Recently, many lantibiotic biosynthetic clusters have been identified using microbial genome database mining approach, by taking advantage of the conserved nature of the genes encoded in the lantibiotic clusters. For example, LanC has been used as a query for the identification of type IA lantibiotic clusters [43], LanM for type IB clusters [30], [44], C39 protease domain for double glycine motif containing precursor peptides [45] and recently HylD and LanT was used for the identification of bacteriocins in cyanobacteria [44]. In the present study, a similar approach was followed to identify novel lantibiotic biosynthetic clusters. HalT transporter of haloduracin biosynthetic cluster was taken as a query to screen the bacterial genomes on NCBI Entrez. HalT has a N-terminal C39 protease and a C-terminal ABC-transporter, for haloduracin precursor peptides. This strategy resulted in the identification of 59 novel LanT homologs in 54 bacterial genomes (more than one in a single genome; **Table 2**). The detailed characterization of all these LanT homologs and the nearby genome sequence, led to the identification of 24 novel lantibiotic clusters, described here. The bacteria encoding these clusters were the representatives of actinobacteria, firmicutes, cyanobacteria, proteobacteria and chloroflexi.

Many examples of actinobacteria have been reported earlier for producing lantibiotics [46]. This study led to the identification of five more actinobacteria (**Fig. 2**) encoding lantibiotic clusters namely *S. hygroscopicus* ATCC 53653, *S. bingchenggensis* BCW-1, *S. viridochromogenes* DSM 40736, *S. roseosporus* NRRL 11379 and *Mycobacterium tusciae* JS617. The three actinobacteria, ATCC 53653, BCW-1 and DSM 40736 were found encoding a putative two-component lantibiotic cluster, as suggested by the phylogenetic analysis of the precursor peptides (**Fig. 3**) and the presence of two LanMs. In ATCC 53653, the ORF encoding a second LanM was missed out in the genomic annotation while in BCW-1, it was hidden and fused with the LanT homolog (**Fig. 4**). It was only the sequence identity among the precursor peptides (7% and 20%), that gave us a clue for the possible presence of a second LanM. To draw a relation between the sequence identity among the precursor peptides and number of LanMs that are required for the processing, examples of already known and putative lantibiotic clusters were taken and analysed (**Table 1**). We could conclude therefrom that for upto 20% sequence identity, two LanMs are required for processing of the precursor peptides and above 37% identity, a single LanM is sufficient.

A majority of known lantibiotics are produced by firmicutes [46]. In this study, we have identified seven strains of *Bacillus cereus* (**Fig. 5**) namely FRI-35, VD107, MSX-A1, SJ1, VD045, VD166 and Rock1–3, encoding novel lantibiotic clusters with a diversity in the sequence of the precursor peptides. Earlier, Xiong *et al* had identified a cluster containing six identical precursor peptides in another strain of *B. cereus* Q1, which were named as cereicidins [36]. Other firmicutes that were identified included the anaerobic bacteria: *R. flavifaciens* FD-1 and *C. bescii* DSM 6725; facultative anaerobe: *S. epidermidis* and aerobic bacteria: *C. perfringens, Bacillus* Str. ATCC 3626, *Bacillis* sp. 7_6_55CFAA_CT2. The strains FRI-35, FD-1 and 7_6_55CFAA_CT2 were found encoding a putative two-component lantibiotic cluster. FRI-35 was found to be encoding 8 and FD-1 as 12 precursor peptides, this being maximum in any firmicute identified till date, although multiple precursor peptides encoding clusters have been identified earlier in cyanobacteria [32], [44] and chloroflexi [30]. The lantibiotic clusters identified in the firmicutes, FRI-35 and *S. epidermidis* were found on plasmids, like the previously characterized lantibiotics cytolysin, nisin, salivaricin etc.

Nine of the previously genetically characterized type IB clusters [46] and four of the clusters identified here in actinobacteria, completely rely on a bifunctional LanT. However, in six of the identified firmicutes, an additional LanP protease was found encoded in the cluster, namely in FRI-35, MSX-A1, SJ1, VD045 (**Fig. 5**), 7_6_55CFAA_CT2 and *S. epidermidis* (**Fig. 6**). The presence of two proteases in a single cluster suggests that there might be more than one cleavage site on the same precursor peptide [44]. While LanT processes and transports a precursor peptide with double glycine motif, the LanP is a membrane bound extracellular protease [20], which acts on the N-terminus of the P(QRS) motif of the precursor peptides. The latter motifs were found present in the leader region of the precursor peptides (**Text S1**) before the double glycine motif in the strains FRI-35, MSX-A1 and SJ1 while in VD045, it was present in the core peptide region. In 7_6_55CFAA_CT2 and *S. epidermidis*, the motif was not identified. So, it will be interesting to know how the presence of both the proteases fit into the sequence of events in lantibiotic biosynthesis.

In 2009, Begley *et al* reported the presence of lantibiotic clusters by *in-silico* analysis in the representatives of cyanobacteria, chloroflexi and proteobacteria, which had not been associated with lantibiotic production earlier [30]. Later, it was experimentally confirmed in cyanobacteria [32]. Thereafter, Wang *et al* reported the widespread occurrence of bacteriocin gene clusters in cyanobacteria, suggesting the phyla to be a prolific source of bacteriocins [44]. Here, we have added two more representatives of cyanobacteria; *Synechocystis* sp. PCC 7509 and a new genus *Chamaesiphon minutus* PCC 6605 (**Fig. 7**), both encoding a putative two-component lantibiotic cluster (**Fig. 3**) and one more genus to chloroflexi, the *Ktedonobacter racemifer* encoding a lantibiotic cluster.

To the best of our knowledge, salivaricin is the only known example where the double glycine type precursor peptide is modified by LanBC (instead of LanM), processed and transported by a bifunctional LanT [47]. In our study, similar biosynthetic clusters were identified in an actinomycete, *M. tusciae* JS617 **(** **Fig. 2** **)** and in proteobacteria, *C. coralloides* DSM 2259 and *C. fuscus* DSM 2262 **(** **Fig. 7** **)**. LanBC enzymes usually process the precursor peptides, which have a conserved proline at -2 position and FNLD motif around the position -20 and -15 in the leader, thus playing a crucial role in the post-translational modification [48]. Experimental evidences are therefore required to conclude that without the presence of these crucial motifs, as has been noticed in the above three clusters, the identified precursor peptides could still undergo post-translational modification by LanBC.

Many different kinds of post-translational modifications, other than the lanthionine formation have been reported recently in bacteriocins [41], [49]. In the present study, besides the normal genes present in the lantibiotic cluster, some additional atypical genes were found like NADPH dependent FMN reductase in SJ1, VD045, *C. bescii* DSM 6725 and *C. perfringens* B str. ATCC 3626 and N-acetyltransferases were found in close proximity to the clusters in DSM 6725 and ATCC 53653. These atypical genes might be involved in additional post-translational modifications of the lantibiotics. N-terminal acetylation of proteins is believed to protect the proteins from degradation [50] and, thus, might provide an additional stability to the encoded lantibiotic, besides that by the lanthionine group.

Many of the previous, putatively [30] and experimentally characterized lantibiotics including nisin have been identified on transmissible genetic elements. Here also, the clusters identified in *B. cereus* VD045, *B. cereus* Rock1–3 and *K. racemifer* DSM 44963 were present in close proximity with the transposable genetic elements, which might be involved in mobilizing the cluster to other species and also for their mobility within the genome.

From several studies done on lantibiotics, it has been felt that the search for smaller antimicrobial peptides is difficult because of their small size and low homology, and are therefore missed out during the conventional genomic annotation. However, the relatively larger transporter gene of the lantibiotic biosynthetic cluster i.e. LanT is rarely unannotated and can be identified by BLAST search. The *in silico* analysis of the nearby region for the lantibiotic modification genes can then lead to the identification of the precursor peptides. Thus, identifying lanthipeptides using conserved regions of the larger proteins of the lantibiotic biosynthetic cluster proves out to be a better approach. The putative lantibiotic clusters identified here using the above said strategy is a major expansion to the already reported putative lantibiotics, and if taken for focussed wet lab experiments, it might expand our library of antimicrobial peptides against the drug resistant pathogens. The identified bacteria can be taken up for *in vivo* production of the lantibiotic or an *in vitro* reconstitution approach can be adopted. Some findings like the presence of LanBC modification enzymes for processing peptides with double glycine motif, fusion of the modification enzyme with the transporter and presence of unrelated genes like the FMN reductases and N-acetyltransferases in the lantibiotic biosysnthetic cluster organization has further pointed towards the need of experimental characterization of these lantibiotic biosynthetic systems. The use of BAGEL2 poses limitations in identification of the whole biosynthetic cluster that can be overcome by a consolidation of well established analytical bioinformatic tools used here. In nutshell, our results provide a higher level of confidence in the novel identified clusters, to proceed for wet lab experiments, for the discovery of novel lantibiotic(s).

### Supporting Information

Table S1 **List of all the putative lantibiotic precursor peptides identified in this study.**
(XLSX)

Text S1 **Processing sites present in the precursor peptides, identified in the clusters encoding two proteases, i.e. the C39 protease of the LanT transporter and the S8 peptidase, LanP.**
(DOCX)
