# Supplementary material for: Novel LanT Associated Lantibiotic Clusters Identified by Genome Database Mining
Source: PLoS One. 2014 Mar 12;9(3):e91352. doi: 10.1371/journal.pone.0091352 (PMC3951391; doi:10.1371/journal.pone.0091352)
Supplement: Text S1 — Processing sites present in the precursor peptides, identified in the clusters encoding two proteases, i.e. the C39 protease of the LanT transporter and the S8 peptidase, LanP. (DOCX) [file pone.0091352.s002.docx]

**Text S1. Precursor peptides identified in the clusters, encoding both LanT and LanP.** Double glycine motif is shown in green color and known motifs required for processing by LanP i.e. P(QRS) is shown in blue, underlined.

*Bacillus cereus* FRI-35

>BCK_27598 (Bacillus cereus FRI-35)

MSKNEKLNKLRNQDLDTKELIGLIDENELKKIA**GA**GDVNPEIAPTPHTVTTIKFTTVCPITHSLV

>BCK_27603 (Bacillus cereus FRI-35)

MSKNEKLNKLRNQEFDTKELIGLVDENELKKVA**GA**GDVNPEIMSDPVTLTIVVPIFTNKLCPTVPCTLKG

>BCK_27608 (Bacillus cereus FRI-35)

MSKNEKLNKLRDQEFDTKELIGSVDENDLKQVA**GA**GDVNPETTPATPTIVAVSLGICPTTKCTSKC

>BCK_27613 (Bacillus cereus FRI-35)

MSKNEKLNKLRDQEFDTKELIGSVDENDLKQVA**GA**GDVNPETTPATPTIVAVSLGICPTTKCTSKC

>BCK_27618 (Bacillus cereus FRI-35)

MSKNEKLNKLRDQEFDTKELIGSVDENDLKQVA**GA**GDVNPETTPATPTIVAVSLGICPTTKCTSKC

>BCK_27623 (Bacillus cereus FRI-35)

MSSKKVVESWKNPVLRSKNEDA**PS**HPAGEVDSKEIKELF**GA**GEGDVTPEGLSSWLGNKGGYCTLTKECM**PS**CN

>BCK_27628 (Bacillus cereus FRI-35)

MSSKKVVESWKNPVLRSKNEDA**PS**HPAGEVDSKEIKELF**GA**GEGDVTPEGLSSWLGNKGGYCTLTKECM**PS**CN

>BCK_27633 (Bacillus cereus FRI-35)

MSSKKVVESWKNPVLRSKNEDA**PS**HPAGEVDSKEIKELF**GA**GEGDVTPEGLSSWLGNKGGYCTLTKECMPSCN

*Bacillus cereus* MSX-A1

>II5_05392 (Bacillus cereus MSX-A1)

MVKKFKFTKEELVEAWKD**PQ**VREKLKDLPNH**PS**GKALNELSEEELAEIQ**GA**SDVQPETTPL**C**VGVIIGLTTS

>II5_05393 (Bacillus cereus MSX-A1)

MTKGYKFTKEELVEAWKD**PQ**VREKLKDLPKH**PS**GKALNELSEEELAEIQGASDVQPETTPL**C**VGVIIGLTTSIKI**C**K

>II5_05394 (Bacillus cereus MSX-A1)

MTKGYKFTKEELVEAWKD**PQ**VREKLKDLPKH**PS**GKALNELSEEELAEIQ**GA**SDVQPETTPL**C**VGVIIGLTTSIKI**C**K

>II5_05395 (Bacillus cereus MSX-A1)

MSKEYKFTKEELVEAWKD**PQ**VREKLKDLPKH**PS**GKALNELSEEELAEIQ**GA**SDVQPETTPL**C**VGVIIGITASIKI**C**K

*Bacillus cereus* SJ1

>BCSJ1_09323 [Bacillus cereus SJ1]

MTNEEIIVAWKNPKVRGKNM**PS**H**PS**GVGFQELSINEMAQVT**GG**AVEQRATPATPATPWLIKASYVVSGAGVSFVASYITVN

>BCSJ1_09328 [Bacillus cereus SJ1]

MTNEEIIVAWKNPKVRGKNM**PS**H**PS**GVGFQELSINEMAQVT**GG**AVEQRATPTLATPLTPHTPYATYVVSGGVVSAISGIFSNNKT**C**LG

>BCSJ1_09333 [Bacillus cereus SJ1]

MTNEEIIVAWKNPKVRGKNM**PS**H**PS**GVGFQELSINEMAQVT**GG**AVEQRATPATPATPWLIKASYVVSGAGVSFVASYITVN

*Bacillus cereus* VD045

>IIE_05066 [Bacillus cereus VD045]

MRNLKEDVVGLSMKKLNTLEMEKIY**GA**SGVDTRTHSMAIVATTTTRTVGQTGPVISRVLSLNTI**C**L

>IIE_05067, partial [Bacillus cereus VD045]

MKNISEKSVGLSMKRLDTTEMEKIY**GA**SGVD

>IIE_05068 [Bacillus cereus VD045]

MKNISEKSVGLSMKKLDATEMEKIY**GA**SGVDTRTHPTVVVVSRASSKF**C**VTVAASAVLSYNMNK**C**

>IIE_05069 [Bacillus cereus VD045]

MKNISEKSVGLSMKKLDTTEMEKIY**GA**SGVDTRTHPTVIVVSRASSKA**C**LSGVSAISGLLSYNKD**C**IG

>IIE_05070, partial [Bacillus cereus VD045]

MKNISEKSVGLSMKKLDTTEMEKIY**GA**SGVD**PR**TT**PS**PLLASFVAS

>IIE_05071, partial [Bacillus cereus VD045]

KLDTTEMEKIY**GA**SGVD**PR**TT**PS**PLLASFVASYIASAQYR**C**GKDNKGK

*Bacillus* sp. 7_6_55CFAA_CT2

>HMPREF1014_04854 [Bacillus sp. 7_6_55CFAA_CT2]

MRKYNIIQNWKNPMKKTEVGTIENPVGNIFEEISDTDLQMA**GG**EDASNYSLIIDIDRMSKILGNKGRI**C**TYTLE**C**TAG**C**NLNPNKK

>HMPREF1014_04855 [Bacillus sp. 7_6_55CFAA_CT2]

MKRDDLRKDIEVSRDQIGKVKESELKEMA**GA**GTSNVEPYGIFHSITF**C**FTLEIGNHS**C**YPNSIK**C**

>HMPREF1014_04856 [Bacillus sp. 7_6_55CFAA_CT2]

MNLQKKLTKKQLREQFNFDESIGKVDEAELVELS**GA**SGEVTPYTTWG**C**AIFTIASAAQ**C**PTTA**C**SSK**C**L

*Staphylococcus epidermidis*

>SAP106A_002 [Staphylococcus epidermidis]

MRSNLERNPYLRNEAQQSVDLPLDNPIRELKEDELLKLN**GA**KNRFIPGAEVAVSTLG**C**YGGSVALGNDGFM**C**TTTVE**C**QNQ**C**K
